# NDRG2 inhibition facilitates angiogenesis of hepatocellular carcinoma

**DOI:** 10.1515/med-2021-0268

**Published:** 2021-05-11

**Authors:** Jianlong Wang, Tao Li, Lifeng Ma, Guochao Liu, Guiying Wang, Jiansheng Kang

**Affiliations:** Minimally Invasive Surgery Department of Biliary Duct, The Second Hospital of Hebei Medical University, Shijiazhuang, 050000, Hebei province, China; General Surgical Department, The Third Hospital of Hebei Medical University, Shijiazhuang, 050000, Hebei province, China; General Surgical Department, The Fourth Hospital of Hebei Medical University, Shijiazhuang, 050000, Hebei province, China

**Keywords:** hepatocellular carcinoma, NDRG2, angiogenesis, VEGFA

## Abstract

Hepatocellular carcinoma (HCC) is an angiogenesis-dependent tumor, and angiogenesis plays pivotal roles in progression and hematogenous metastasis. Upregulating NDRG2 expression could inhibit endothelial cell proliferation and tumor angiogenesis. However, the development of angiogenesis is a complicated and dynamic process, and the specific mechanisms that NDRG2 influences its progression are largely unknown. Conditioned media (CM) was collected from HCC cells. Cell viability, migration assay, tube formation, and western blot were used to evaluate the effect of NDRG2 on angiogenesis in HCC cells. ELISA assay was used to measure the level of VEGFA in CM. CM from NDRG2 knockdown cells significantly promoted HUVECs proliferation, migration, and tube formation compared with control cells. The level of VEGFA in CM was increased by NDRG2 knockdown relative to the control group. The expression of VEGFA, HIF-1α, and p-Akt was significantly increased in NDRG2 knockdown cells. CM from NDRG2 knockdown cells with VEGFA antibody failed to induce HUVEC proliferation, migration, and tube formation. YC-1 significantly inhibited the level of VEGFA in CM from NDRG2 knockdown cells. YC-1 also inhibited the expression of VEGFA and HIF-1α. Therefore, NDRG2 inhibition promoted the angiogenesis of HCC via VEGFA and may be used to be an anti-angiogenesis target.

## 1 Introduction

Hepatocellular carcinoma (HCC) is the fourth cause of cancer-related death and a leading cause of death among patients with cirrhosis [1]. Despite improvements in the cancer treatment, the 5-year survival in population with HCC is only 18% [2]. Angiogenesis plays an important role of promoting HCC development, and cancer cells could secret proangiogenic growth factors, which further stimulate the tumor growth [3]. Therefore, strategies targeting angiogenesis are efficient for its protective effects against HCC emergence and progression.

*N-Myc downstream-regulated gene 2* (*NDRG2*) is a newly identified differentiation-related gene that belongs to the NDRG family comprising four members, *NDRG1–4* [4]. Recent studies demonstrated that *NDRG2* functions as a tumor suppressor and may be a prognostic predictor for many different malignant tumors [5,6,7,8,9,10]. Upregulating *NDRG2* expression could inhibit endothelial cell proliferation and tumor angiogenesis in breast cancer cells [10]. Overexpression of *NDRG2* in tumors suppressed the intratumoral and peritumoral angiogenesis in melanoma [11]. However, the development of angiogenesis is a complicated and dynamic process, and the specific mechanisms by which *NDRG2* influences its progression are largely unknown.

The vascular endothelial growth factor (VEGF) family, consisting of VEGF-(A–D), is one of the best characterized and vital groups of protein factors [12]. VEGFA is principally responsible for vessel formation in adult tissues, which binds with higher affinity to VEGFR than the primary receptor involved in endothelial cell proliferation and migration [13]. The first anti-angiogenic agent approved for use was bevacizumab (approved in 2006; Avastin^®^; Genentech Inc., San Francisco, CA) [14]. However, the relationship between NDRG2 and VEGFA in the development of HCC has not been fully delineated.

In the present study, we examined the role of NDRG2 in the angiogenesis of HCC. The effect of condition media from NDRG2 knockdown HCC cell lines was used to evaluate the role of NDRG2 in proliferation, migration, and tube formation of HUVECs. HIF-1α inhibitor and VEGFA antibody were used to investigate the effect of VEGFA in NDRG2-promoting angiogenesis of HCC. We demonstrated that NDRG2 inhibition promoted the angiogenesis in HCC cell lines through VEGFA, and NDRG2 merits further investigation as a promising gene therapy target to treat HCC.

## 2 Materials and methods

### 2.1 Cell culture

The human HCC cell lines, HepG2 and Hep3B, were obtained from the China Center for Type Culture Collection and Cell Bank of the Chinese Academy of Sciences (Shanghai, China), and HUVECs were bought from ScienCell (San Diego, CA). HepG2 and Hep3B cells were maintained in DMEM (Gibco, Thermo Fisher Scientific, Waltham, MA) supplemented with 10% fetal bovine serum (FBS; Gibco, Thermo Fisher Scientific), 2 mM L-glutamine, and 100 U/mL penicillin/streptomycin mixtures. HUVECs were cultured in the endothelial cell growth medium (ScienCell). All cells were cultured in a humidified atmosphere of 5% CO_2_ at 37℃.

### 2.2 Cell viability assay

Cell Counting Kit-8 (Beyotime, Shanghai, China) was used to determine the cell viability. Briefly, 3,000 cells were seeded per well in a 96-well plate and were cultured for 48 h. At the end of the incubation, CCK-8 was added to each well and incubated at 37°C for 4 h. Absorbance was detected at 450 nm by an enzyme-linked immunosorbent assay (ELISA) reader.

### 2.3 Migration assay

Conditioned media (CM) was collected from NDRG2 knockdown cells and control cells. Then, HUVECs were trypsinized, and 2 × 10^4^ cells in 200 μL CM were seeded in the upper chamber of Transwell plates (Corning, NY). A total of 600 μL medium containing 10% FBS was added into the lower chambers. After 24 h culture, the nonmigrating cells in the upper chamber were gently removed, while the migrating cells were stained with 0.1% crystal violet (Solarbio, Beijing, China) for 20 min, washed with water, air dried, and photographed using an Olympus microscope imaging systems. The migrating cells were counted and averaged from images of five random fields for the differential analysis.

### 2.4 Tube formation

Serum-reduced Matrigel (10 mg/mL; BD, San Jose, CA) was thawed overnight at 4℃, and 70 μL was added to each well of a 96-well plate and allowed to solidify for 30 min at 37℃. Wells were incubated with 1 × 10^4^ HUVEC cells for 4 h. Capillary tube formation was observed. The total length and the number of junctions of the tubes were quantified by the analysis of digitized images using ImageJ software and the Angiogenesis Analyzer plugin of the capillary-like structures.

### 2.5 Gene transfection

Lentiviral vectors containing targeted sequences were constructed in GenePharma (Shanghai, China). The sequences were displayed as flowed:

NDRG2: forward, 5′-CCGGGAGGACATGCAGGAAATCATTCTCGAGAATGATT-TCCTGCATGTCCTCTTTTTG-3′, reverse, 5′-AATTCAAAAAGAGGACATGCA-GGAAATCATTCTCGAGAATGATTTCCTGCATGTCCTC-3′. Scramble: forward, 5′-CCGGAAGGTCTTGTCCTCATCAACACTCGAGTGTTGATGAGGACAAGA-CCTTTTTTTG-3′, reverse, 5′-AATTCAAAAAAAGGTCTTGTCCTCATCAACAC-TCGAGTGTTGATGAGGACAAGACCTT-3′. HepG2 and Hep3B cells were transfected with lentiviral vectors including the shNDRG2 or shscramble expression using lipofectamine 3000. After 48 h, HepG2 and Hep3B cells were cultured with DMEM containing puromycin (2 μg/mL). The expanded cells were then used for further experiments. Transduction efficiency was confirmed by the western blot.

### 2.6 Conditioned media collection

HepG2 or Hep3B cells were transfected with the NDRG2 shRNA or scramble shRNA and then incubated in serum-free DMEM for 24 h followed by collection of the CM. The medium was spun down at 2,500 rpm for 5 min to pellet any debris, and the supernatant was collected and stored at −80℃ until needed.

### 2.7 ELISA assay

The concentration of VEGFA in the CM was determined by ELISA according to the human ELISA kit instructions (Boster Biotechnology, Wuhan, China).

### 2.8 Western blot analysis

Total protein was extracted using RIPA buffer containing protease and phosphatase inhibitors. The protein samples were heated at 100℃ for 10 min, separated by sodium dodecyl sulfate-polyacrylamide gel electrophoresis (SDS-PAGE), and transferred onto PVDF membranes (EMD Millipore, Billerica, MA). The membranes were blocked in 5% bovine serum albumin (BSA) at room temperature for 1 h. Immunoblotting was performed with antibodies against β-actin, VEGFA, p-Akt, t-Akt, and HIF-1α (Abcam, Cambridge, MA). Then, the blot was detected with horseradish peroxidase-labeled secondary antibody. Signals were visualized using the ECL detection system (Millipore, Boston, MA) according to the manufacturer’s instructions.

### 2.9 Statistical analysis

The data are expressed as the mean ± SD. Data analyses were performed with GraphPad Prism5 (GraphPad Software, La Jolla, CA). The comparison between two groups was done using Student’s *t*-test. Multiple group variables with similar variance were performed by two-way analysis of variance (ANOVA). A *P* value of <0.05 was considered statistically significant.

## 3 Results

### 3.1 NDRG2 inhibition promotes angiogenesis of HCC *in vitro*

**Figure 1:**
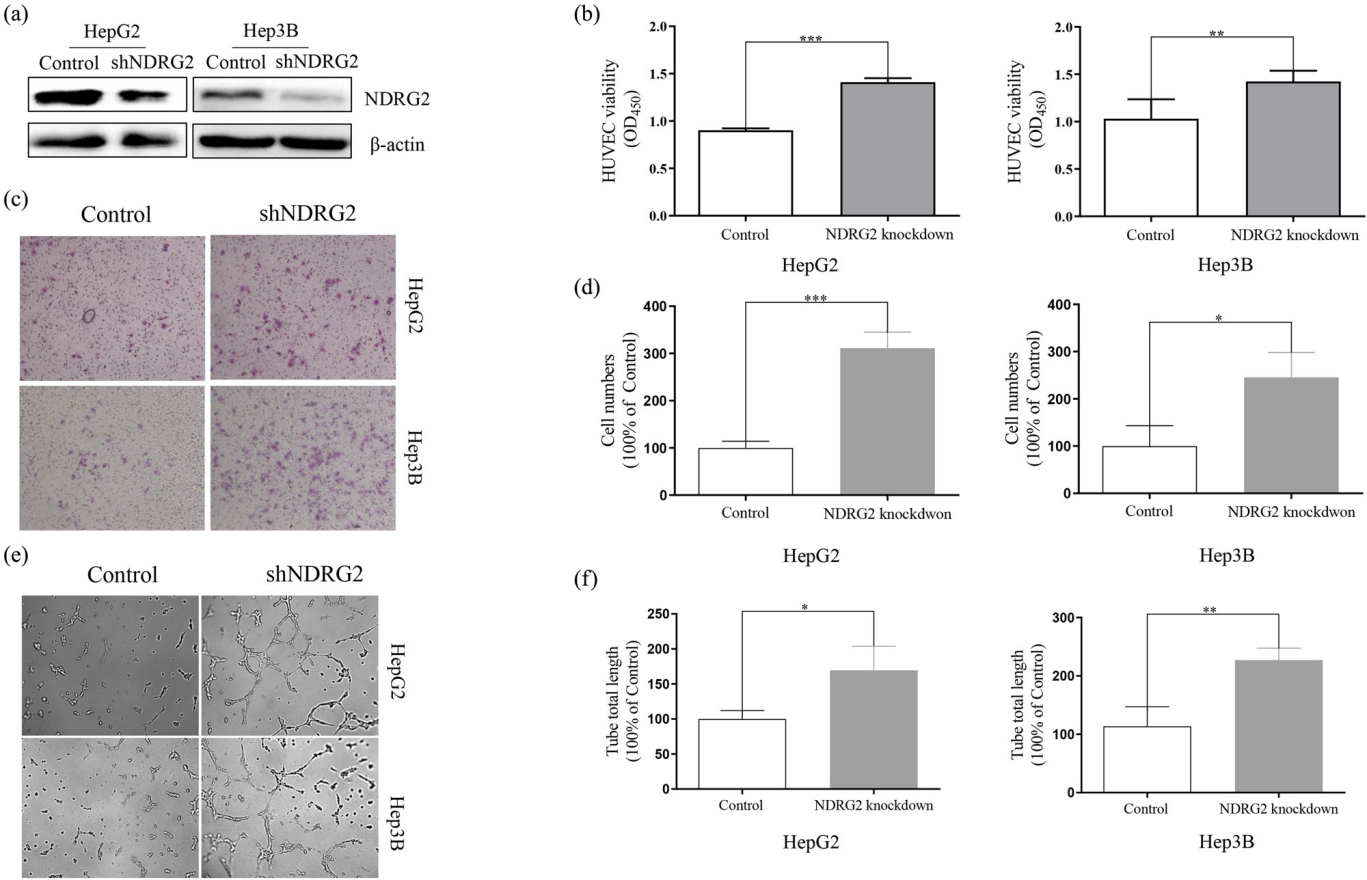
NDRG2 inhibition promoted angiogenesis of HCC *in vitro*. We knocked down NDRG2 expression in HepG2 and Hep3B cells and collected the CM. CM was used to treat HUVECs. (a) NDRG2 was knocked down in HepG2 and Hep3B by shRNA. The results were confirmed by the western blot. CM from NDRG2 knockdown cells significantly promoted HUVECs proliferation (b), migration (c and d), and tube formation (e and f) compared with control cells. ^*^ *P <* 0.05; ^**^ *P <* 0.01; ^***^ *P <* 0.001.

We investigated whether NDRG2 acted in a paracrine manner to regulate tumor angiogenesis using HepG2 and Hep3B cells. HUVEC proliferation, migration, and tube formation were evaluated using the CM from HepG2 and Hep3B cells. We knocked down NDRG2 expression in HepG2 and Hep3B cells, and the results were confirmed by the western blot (Figure 1a). CM from NDRG2 knockdown cells significantly promoted HUVEC proliferation compared with that from control cells (Figure 1b). Meanwhile, HUVECs treated with CM from NDRG2 knockdown cells showed enhanced migratory ability compared with that from the control cells (Figure 1c and d). Furthermore, the longer tube total length was found in HUVECs treated with CM from NDRG2 knockdown cells compared with that from control cells (Figure 1e and f).

### 3.2 NDRG2 may be involved in the angiogenesis of HCC via VEGFA

**Figure 2:**
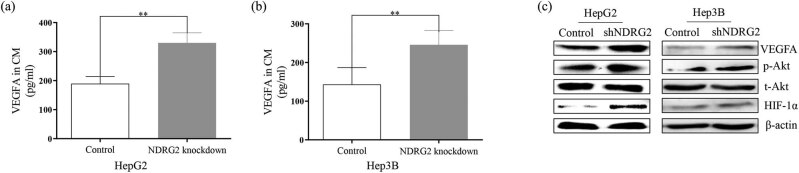
NDRG2 knockdown increased the level of secretory VEGFA. We examined the changes of VEGFA levels in CM from HepG2 and Hep3B cell culture following NDRG2 knockdown using ELISA. (a–b) The level of VEGFA was increased by NDRG2 knockdown relative to the control group. (c) The expression of VEGFA, HIF-1α, and p-Akt were upregulated in NDRG2 knockdown cells compared with control cells. ^**^ *P <* 0.01.

We examined changes in VEGFA levels in CM from HepG2 and Hep3B cells following NDRG2 knockdown using ELISA. The level of VEGFA was increased by NDRG2 knockdown relative to the control group (Figure 2a and b). Meanwhile, the expression of VEGFA was upregulated in NDRG2 knockdown cells compared with control cells (Figure 2c). We also examined the associated signaling involved in the process. We found that the expression of HIF-1α and p-Akt was significantly increased in NDRG2 knockdown cells (Figure 2c). The results indicated that p-Akt/HIF-1α may be involved in the NDRG2-inducing VEGFA upregulation.

**Figure 3:**
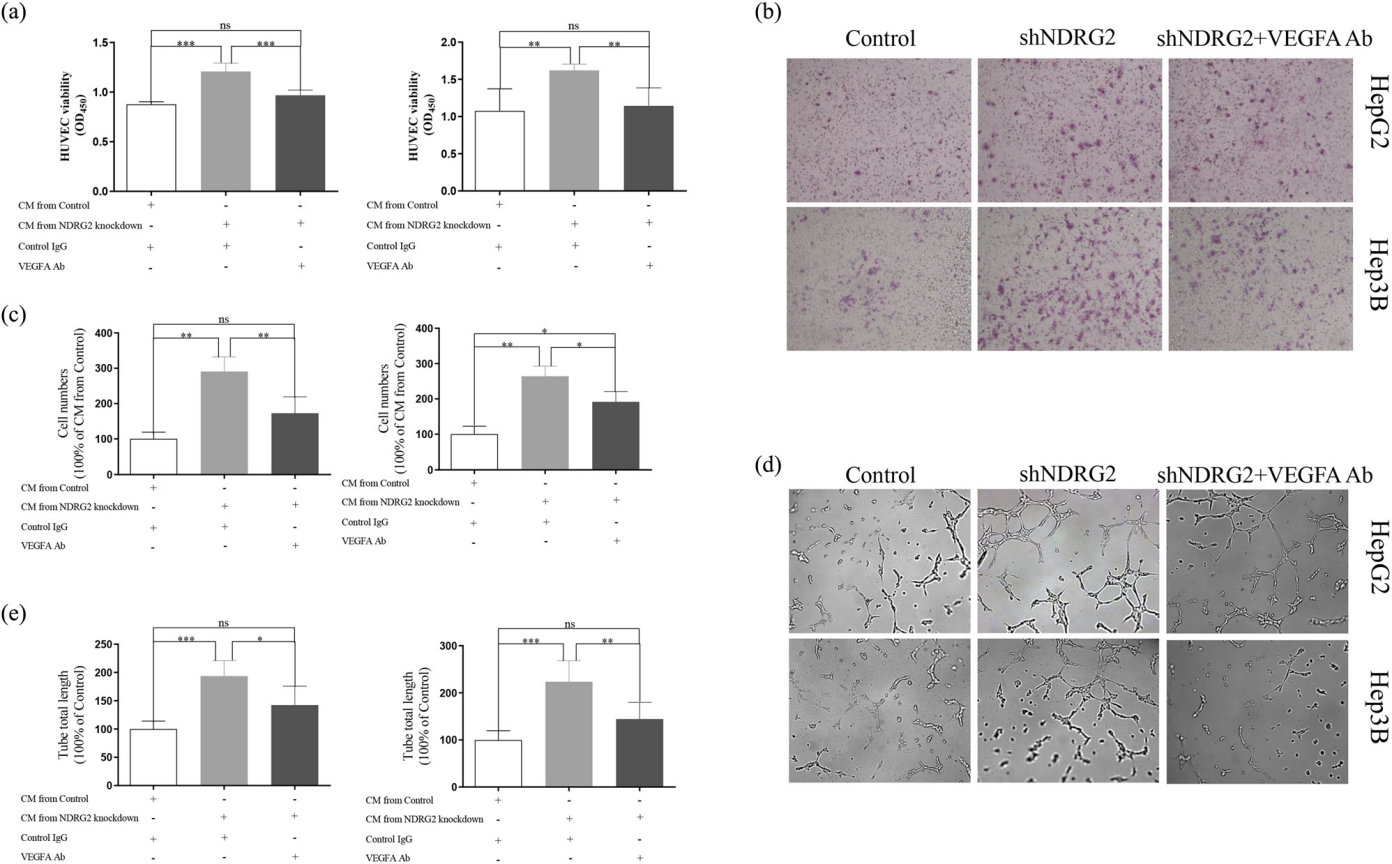
VEGFA may be involved in the NDRG2-knockdown promoting angiogenesis in HepG2 and Hep3B cells. VEGFA antibody was added to the CM. CM from NDRG2 knockdown cells with VEGFA antibody failed to induce HUVECs proliferation (a), migration (b and c), and tube formation (d and e). ns, no significance; ^*^ *P <* 0.05; ^**^ *P <* 0.01; ^***^ *P <* 0.001.

To confirm whether VEGFA was involved in the NDRG2-induced angiogenesis of HCC, VEGFA antibody was added to the CM. After adding the VEGFA antibody, CM from NDRG2 knockdown cells failed to induce enhanced HUVECs proliferation, and the difference between CM from NDRG2 knockdown cells with VEGFA antibody and CM from control cells was eliminated (Figure 3a). Furthermore, HUVECs treated with CM from NDRG2 knockdown cells with VEGFA antibody showed impaired migratory ability compared with that from NDRG2 knockdown cells with control IgG (Figure 3b and c). The difference between CM from NDRG2 knockdown cells with VEGFA antibody and CM from control cells in HepG2 cells was eliminated, and the difference between CM from NDRG2 knockdown cells with VEGFA antibody and CM from control cells in Hep3B cells was shrunk (Figure 3b and c). Meanwhile, the longer tube total length was found in HUVECs treated with CM from NDRG2 knockdown cells with control IgG compared with that from NDRG2 knockdown cells with VEGFA antibody (Figure 3d and e). The difference between CM from NDRG2 knockdown cells with VEGFA antibody and CM from control cells was eliminated (Figure 3d and e)

### 3.3 HIF-1α is needed in the NDRG2-VEGFA inducing angiogenesis

**Figure 4:**
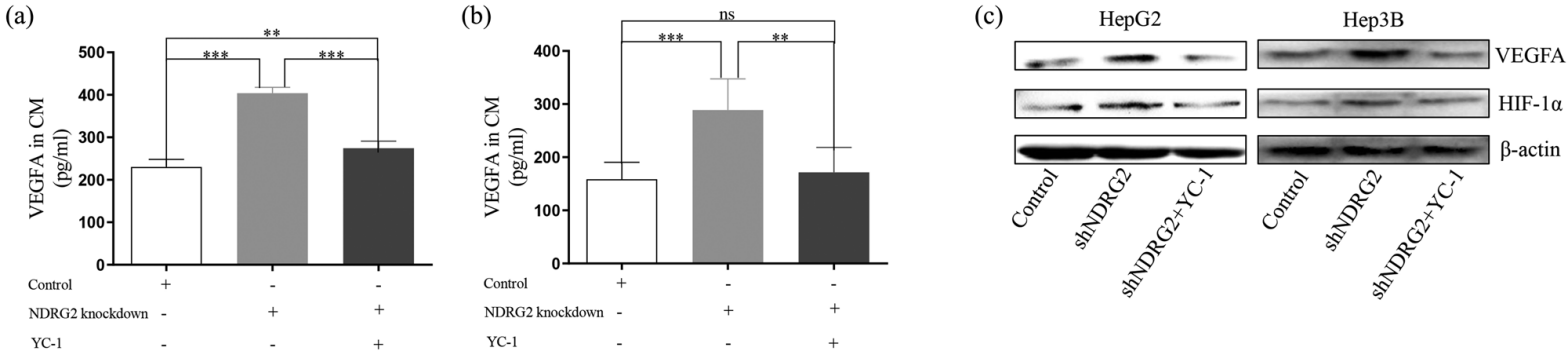
HIF-1α is needed in the NDRG2-VEGFA inducing angiogenesis. HIF-1α inhibitor YC-1 was used to evaluate the effect of NDRG2 on the VEGFA expression. (a–b) YC-1 significantly inhibited the levels of VEGFA in CM from NDRG2 knockdown cells. (c) That YC-1 inhibited the expression of VEGFA and HIF-1α was confirmed by the western blot. ns, no significance; ^**^ *P <* 0.01; ^***^ *P <* 0.001.

HIF-1α inhibitor, YC-1, was used to evaluate the signaling pathway participating in NDRG2 inducing VEGFA expression. We found that YC-1 significantly inhibited the levels of VEGFA in CM from NDRG2 knockdown in HepG2 and Hep3B cells (Figure 4a and b). The difference of VEGFA concentration between CM from NDRG2 knockdown cells with YC-1 treatment and control cells in HepG2 cells was shrunk, and the difference in Hep3B cells was eliminated. Meanwhile, YC-1 inhibited the increased expression of VEGFA and HIF-1α in NDRG2 knockdown cells (Figure 4c).

## 4 Discussion

HCC is an angiogenesis-dependent tumor, and angiogenesis plays pivotal roles in progression and metastasis [15]. Whether NDRG2 could be involved in angiogenesis of HCC has not been demonstrated. In the present study, we provided additional evidence showing the involvement of NDRG2 in regulation of angiogenesis in HCC. NDRG2 knockdown could promote the proliferation, migration, and tube formation of HUVECs, and HIF-1α/VEGFA may be involved in the process.

Angiogenesis is a complex process by which new blood vessels are formed from endothelial precursors, which is a critical determinant in tumor initiation, progression, and metastasis [16,17]. Upregulating NDRG2 expression inhibited the proliferation and tumor angiogenesis in breast cancer [18]. Overexpression of NDRG2 in tumor suppressed the intratumoral and peritumoral angiogenesis in melanoma [11]. We also found that CM from NDRG2 knockdown cells promoted the proliferation, migration, and tube formation of HUVECs in HCC cells. Therefore, NDRG2 may be an anti-angiogenesis target to prevent the progression of HCC. VEGFA could lead to increased vascular permeability and plays a crucial role in physiological and pathological angiogenesis [19]. VEGF overexpression in HCC indicated a higher risk of recurrence, metastasis, and death [20,21]. Bevacizumab, a monoclonal antibody targeting VEGFA, shows considerable PFS and OS improvement in combination with atezolizumab compared to that with sorafenib in unresectable HCC [22]. VEGFA also induces the endothelial cell division and migration, promotion of endothelial cell survival through protection from apoptosis, and reversal of endothelial cell senescence [23]. In our study, we found that the level of VEGFA was increased by NDRG2 knockdown relative to the control group, and CM from NDRG2 knockdown cells with VEGFA antibody failed to induce HUVEC proliferation, migration, and tube formation. The results suggested that VEGFA was involved in the NDRG2 silence–promoting angiogenesis in HCC.

Hypoxia inducible factor 1α (HIF-1α) plays a key role in tumor angiogenesis and regulates the expression level of VEGF [24]. HIF-1α also could result in the increased expression of various genes involved in diverse biological functions under normoxia, including cell proliferation, apoptosis, migration, invasion, and angiogenesis [25]. The upregulated expression of HIF-1α is associated with a poor prognosis and worse overall survival in HCC patients [26,27]. Overexpression of NDRG2 upregulated the expression of pVHL along with downregulation of HIF-1α and attenuated proliferation and invasion in renal cancer cells [28]. In our study, HIF-1α was upregulated in NDRG2 knockdown cells under normoxia, and YC-1 inhibited the level and expression of VEGFA in NDRG2 knockdown cells, which suggested that HIF-1α was involved in the NDRG2 knockdown promoting VEGFA expression. PI3K/Akt plays crucial roles in endothelial cell survival and angiogenesis in HCC [29,30]. NDRG2 also has a growth inhibitory effect on several malignant cell lines through the PI3K/Akt pathway [31,32]. In the present study, the expression of p-Akt was increased in the NDRG2-knockdown cells, which indicated that PI3K/Akt signaling may be involved in the NDRG2 inhibition, promoting angiogenesis of HCC.

In summary, NDRG2 knockdown promoted the angiogenesis of HCC and may be used to be an anti-angiogenesis target. Our results provided the first *in vitro* evidence that NDRG2 may be involved in HCC angiogenesis by regulating VEGFA secretion, which may indicate the future direction for the development of targeted drugs.
